# Tracking the Complex Dynamics of Electron-Transfer-Mediated Decay in Real Space and Time

**DOI:** 10.1021/jacs.5c15510

**Published:** 2026-01-22

**Authors:** Florian Trinter, Jaroslav Hofierka, Jonas Rist, Max Kircher, Miriam Weller, Niklas Melzer, Dimitrios Tsitsonis, Angelina Geyer, Jan Kruse, Gregor Kastirke, Joshua B. Williams, Tsveta Miteva, Reinhard Dörner, Markus S. Schöffler, Maksim Kunitski, Nicolas Sisourat, Lorenz S. Cederbaum, Till Jahnke

**Affiliations:** † Molecular Physics, 28259 Fritz-Haber-Institut der Max-Planck-Gesellschaft, 14195 Berlin, Germany; ‡ Theoretische Chemie, Physikalisch-Chemisches Institut, 9144 Universität Heidelberg, 69120 Heidelberg, Germany; § Institut für Kernphysik, 54239 Goethe-Universität Frankfurt, 60438 Frankfurt am Main, Germany; ∥ Department of Physics, University of Nevada, Reno, Nevada 89557, United States; ⊥ Laboratoire de Chimie Physique—Matière et Rayonnement (LCPMR), UMR 7614, CNRS, 9173 Sorbonne Université, 75005 Paris, France; # European XFEL, 22869 Schenefeld, Germany; ∇ Max-Planck-Institut für Kernphysik, 69117 Heidelberg, Germany

## Abstract

When an electronically excited atom or molecule is embedded in a chemical environment as, e.g., in a liquid or a loosely bound cluster, it can de-excite through mechanisms where neighboring atoms or molecules are actively participating in the decay: either by donating or accepting energy or electrons. For such *nonlocal* decay channels, nuclear dynamics play a crucial role as they have a direct impact on the decay efficiency itself. Here, we present a detailed study of the electron-transfer-mediated decay in a loosely bound triatomic prototype system, combining experimental results from a 5-fold coincidence measurement and theoretical modeling of the decay process. Depending on the decay time, we find that certain classes of molecular geometries are favored for this type of decay. Our findings provide an intuitive picture of how electron-transfer-mediated decay proceeds. In particular, our results confirm a roaming-like behavior of the atoms of the trimer prior to its decay. Our combined theoretical and experimental approach enables a comprehensive tracing of the real-space properties of the decaying system in the time domain.

## Graphical abstract

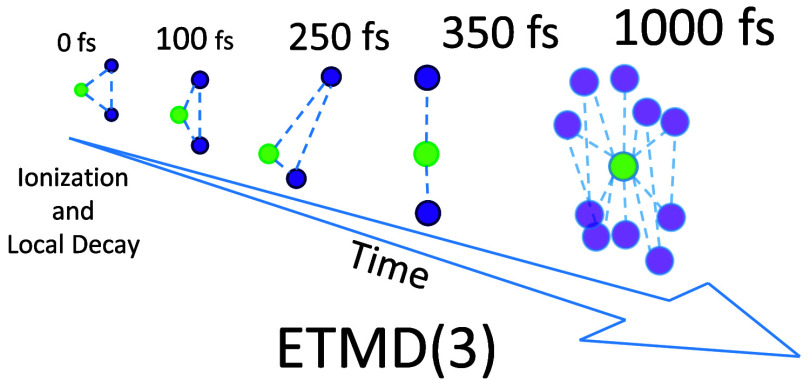

## I Introduction

In the late 20th century, Cederbaum et al. found that in loosely bound matter efficient nonlocal electronic decay processes can occur. In their work, they identified a general mechanism in which the energy freed by the electronic de-excitation of one molecule of a weakly bound cluster is transferred to a neighboring molecule, triggering its ionization. They termed this process interatomic Coulombic decay (ICD).^1^ Since then, several associated processes have been predicted and observed.^2^ Electron-transfer-mediated decay (ETMD)^3^ is one such example. In ETMD, an ionized or excited atom of the loosely bound compound (e.g., a van der Waals cluster) de-excites as a neighboring atom donates an electron. The energy released in this electron transfer leads to further ionization of the electron donor in a so-called “ETMD(2)”.^3–5^ Alternatively, in larger clusters, the energy can be used to ionize a third atom or molecule of the compound in an “ETMD(3)” process.^6–10^ Numerous studies have identified and explored the process in various settings over the past decade.^11–23^ ETMD is of particular importance in the context of radiation damage to biological matter, as it was predicted to efficiently generate reactive species in aqueous environments.^24^ These predictions have recently been confirmed by experiments.^26–28^ With respect to large systems of biological relevance, however, detailed *ab initio* calculations of ETMD dynamics for, e.g., DNA-sized systems are not yet feasible. The increased molecular complexity introduces additional channels, such as proton transfer and nonadiabatic couplings (see, e.g., ref 25). Similarly, corresponding coincidence experiments on such systems remain beyond the current state of the art. ETMD can be triggered by a direct ionization process or as one step in an electronic decay cascade.^29,30^ The existence of the ETMD(3) process, for example, after ionization of the neon 1s shell was initially demonstrated by You et al. in large NeKr mixed clusters.^30^

The typical decay times of excited states that undergo ETMD are in the range of picoseconds. Such long decay times open up the possibility for the excited compound to undergo a significant nuclear rearrangement, which in turn alters the efficiency of the decay. The intricate ETMD dynamics were theoretically studied in a prototypical system of a van der Waals bound NeKr_2_ trimer.^29^ There, based on the assumption that the dynamics preceding ETMD preserves the original symmetry, only symmetric trimer geometries were considered. In fact, the calculations confirmed that the change in the geometry of the trimer alters the probability of ETMD, resulting in a strongly time-dependent decay efficiency. Depending on the exact geometrical arrangement of the three atoms of the trimer, the decay rate varies by almost an order of magnitude and for some geometries the de-excitation is even energetically forbidden. In NeKr_2_, an initial K-shell ionization of the Ne atom of the trimer and a subsequent Auger–Meitner decay result in the population of a doubly charged trimer with two vacancies in the neon atom

1 $${\mathrm{NeKr}}_{2}+h\nu \to {\mathrm{Ne}}^{+}\left(1s^{-1}\right){\mathrm{Kr}}_{2}+e_{\mathrm{photo}}^{-}$$

2 $${\mathrm{Ne}}^{+}\left(1s^{-1}\right){\mathrm{Kr}}_{2}\to {\mathrm{Ne}}^{++}{\mathrm{Kr}}_{2}+e_{\mathrm{Auger}}^{-}$$

Subsequently, the doubly charged trimer can undergo ETMD(3) within picoseconds resulting in the emission of a slow ETMD electron

3 $${\mathrm{Ne}}^{++}{\mathrm{Kr}}_{2}\to {\mathrm{Ne}}^{+}{\left({\mathrm{Kr}}^{+}\right)}_{2}+e_{\mathrm{ETMD}}^{-}$$

4 $${\mathrm{Ne}}^{+}{\left({\mathrm{Kr}}^{+}\right)}_{2}\to {\mathrm{Ne}}^{+}+{\mathrm{Kr}}^{+}+{\mathrm{Kr}}^{+}$$

In Figure 1, the course of events (eqs 1–4) from the initial photoionization (A) to the nonlocal electronic decay of the trimer (D) and its subsequent Coulomb explosion (E) is sketched. Note that we use the term “nonlocal electronic decay” in the standard ICD/ETMD sense: energy or electron transfer between distinct centers within a weakly bound assembly, in contrast to local Auger-Meitner decay. Interestingly, in most cases, the doubly charged trimer in step (B) remains a stable entity until ETMD finally sets in. However, since the compound is loosely bound, its geometry changes during this time (C). Finally, in step (D), ETMD(3) occurs as one krypton atom donates an electron to the doubly charged neon atom, transferring the released energy to the other krypton atom. After this decay, each of the three atoms of the trimer is singly charged, and the trimer fragments rapidly in a Coulomb explosion (E). Figure 2 shows a sketch of the geometry of the trimer in its ground state. It is a (floppy) almost equilateral triangle with the distance between the neon atom and each krypton atom being slightly shorter than the distance between the two krypton atoms. The latter internuclear distance is *R*_Kr–Kr_ = 4.07 Å, the former is *R*_Ne–Kr_ = 3.68 Å, and the angle θ at the neon atom has a value of 67°.^29^

**Figure 1:**
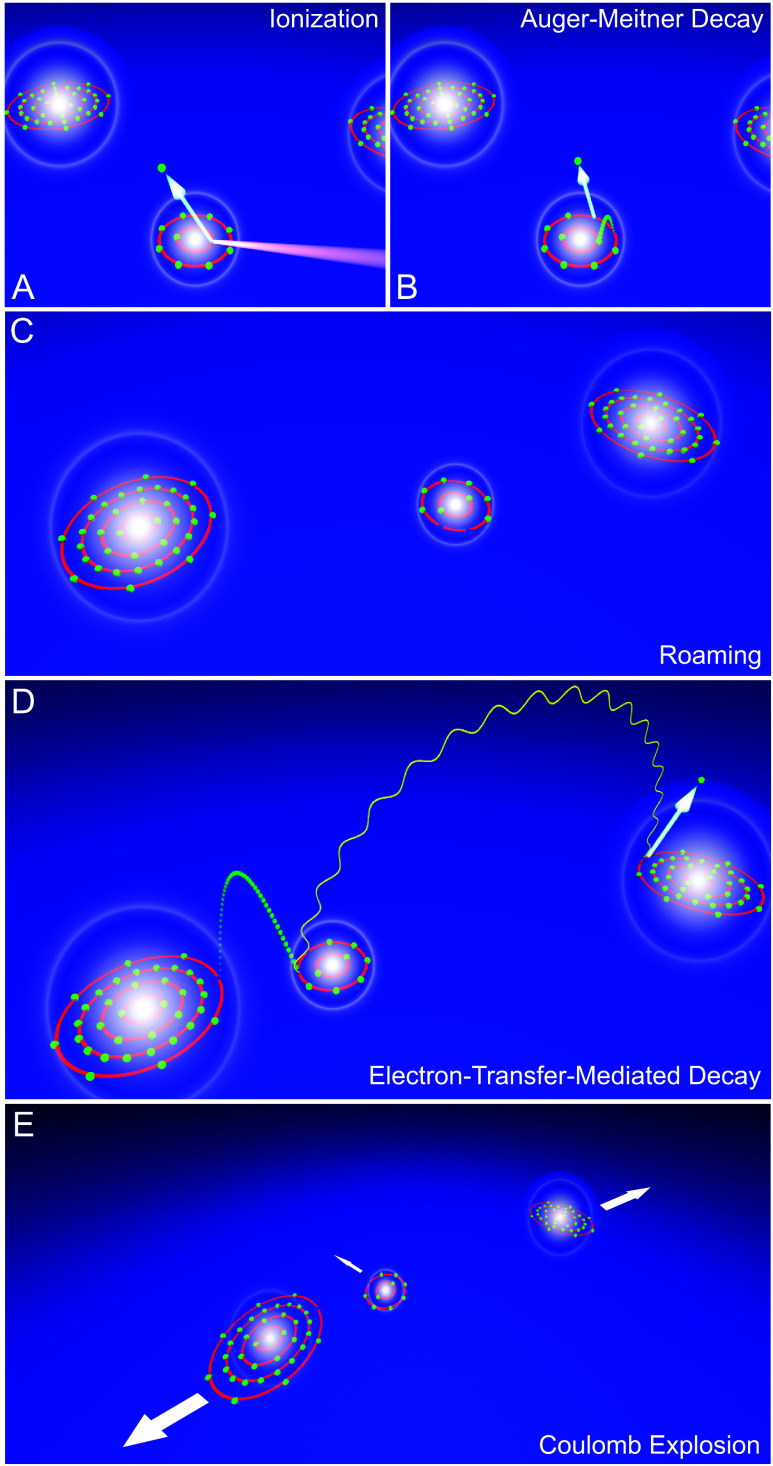
(A) NeKr_2_ trimer is core ionized, and (B) a subsequent Auger–Meitner decay generates a doubly charged neon ion within a few femtoseconds. (C) The ionized trimer remains a stable entity, with its atoms roaming around each other for up to 1 ps. (D) ETMD takes place as one krypton atom donates an electron which fills one of the vacancies in the neon ion. The energy released by this donation is transferred to the other krypton atom and ionizes it. (E) After ETMD, a single charge is located at each atom, leading to a Coulomb explosion of the trimer.

**Figure 2:**
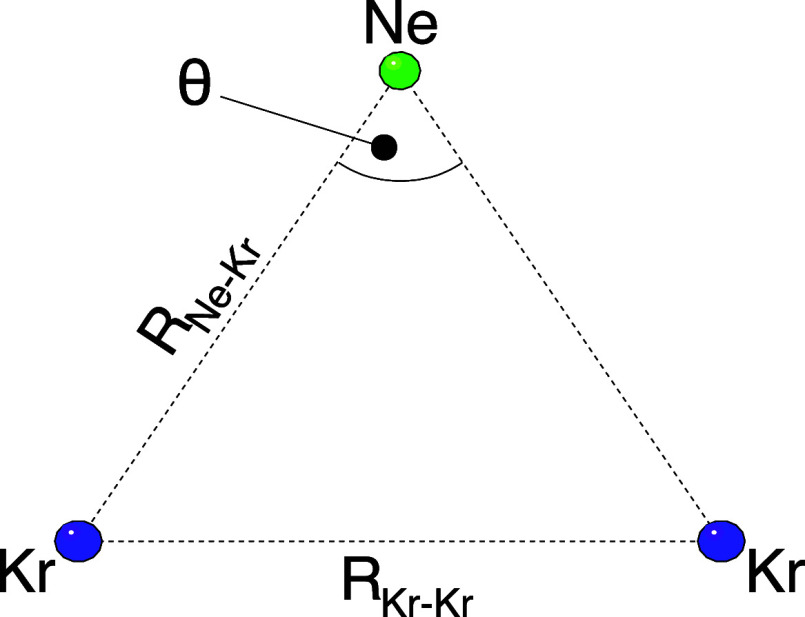
Sketch of the geometry of the trimer in its ground state, see text for mean internuclear distances and angles.

In spite of the accumulated knowledge mentioned above, details on the intricate dynamics prior to ETMD are sparse. In the following, we provide an extensive study of the complex dynamics of ETMD in a trimer, i.e., the smallest entity that allows ETMD(3). Using the coincident detection of ions and electrons generated in the ETMD(3) process with a COLTRIMS reaction microscope^31–33^ in combination with a fully dimensional theoretical modeling of the decay process, we can precisely trace the full temporal evolution of the decay in real space. We observe a pendular, almost roaming-like motion of the trimer’s atoms prior to the de-excitation (see the Supporting Information for a movie visualizing a corresponding trajectory).

## II Ion-Electron Coincidence Results and Discussion

In a first step, we identify events in which the decay process occurred in our experiment. To this end, Figure 3 shows our measured results in the form of a coincidence map that depicts the kinetic energy of one of the measured electrons together with the sum of the kinetic energies of the ionic fragments [kinetic energy release (KER)]. Since the total kinetic energy of the ions and the electron generated by ETMD is fixed and determined by the electronic configuration of the initial and final states, events where the ETMD electron and the three ions have been measured in coincidence (requiring a true five-particle coincidence) appear along diagonal lines with a slope of −1 in Figure 3. After the Auger–Meitner decay of the K-shell vacancy of the neon atom, several states with different electronic structures may be populated. The main contribution consists of two vacancies in the 2p shell of the neon atom in one of its three possible configurations, ^1^S, ^1^D, or ^3^P.^34,35^ Contributions to these states (which then undergo ETMD) occur in Figure 3 along the diagonal lines labeled 1, 2, and 3, respectively. The contribution of the ^1^D state is known to be the strongest with a fraction of approximately 60% ^30,34,36,37^ and is directly visible as a diagonal shape in the range 10 eV < KER < 16 eV (extending the line labeled 2). The photoelectrons from the initial K-shell ionization of the neon atom are located at an energy of approximately 10 eV as indicated by the horizontal dashed line. The bright feature close to zero electron energy results from a different de-excitation pathway where one of the two krypton atoms is initially ionized instead of the neon atom. After a corresponding Auger–Meitner decay, several states are populated as in the neon case. The low-energy feature most likely corresponds to a case in which the ionized and excited system undergoes interatomic Coulombic decay^1^ followed by radiative charge transfer.^38,39^ For our detailed study on ETMD(3), we neglect this and other contributions and focus in the following only on the signal from line 2, the most strongly populated by neon Auger–Meitner decay.

**Figure 3:**
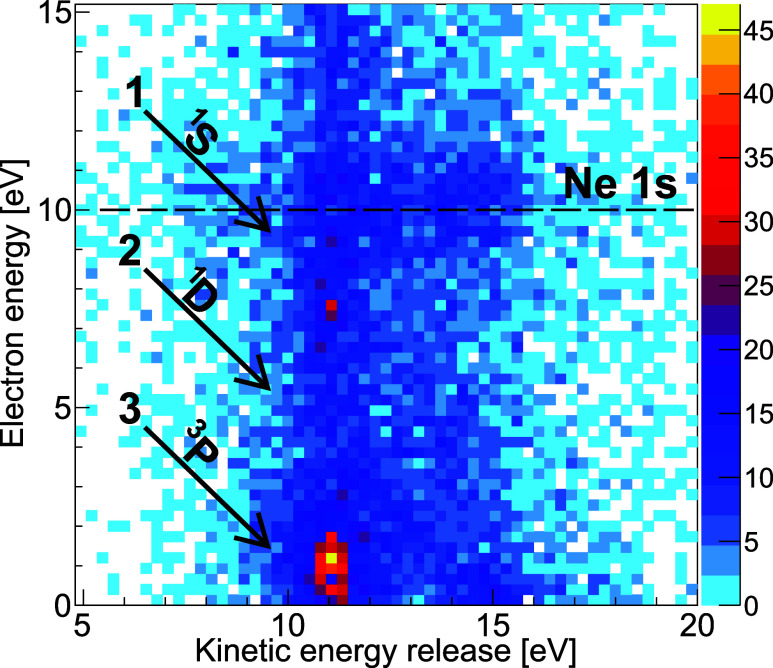
Electron energy as a function of the kinetic energy release (KER, sum of the kinetic energies of the Ne^+^ and the two Kr^+^ ions measured in coincidence with the electron). The Ne K-shell photoelectron is located at an energy close to 10 eV as indicated by the horizontal dashed line. The diagonal feature labeled 2, most strongly populated by Auger–Meitner decay, is visible in the lower third of the panel and belongs to cases where ETMD(3) occurred after the decay of Ne^2+^(2p^–2^) ^1^D Kr_2_. The brightest feature close to zero electron kinetic energy is caused by a different decay route involving an initial photoionization of one of the two krypton atoms (see text). The color scale shows the number of measured counts.

The Coulomb repulsion between the three singly charged ions in the final state after ETMD(3) leads to a fragmentation of the trimer in a “Coulomb explosion”. By measuring the momenta of the ions after the explosion, we can extract information on the geometry of the trimer at the instant of ETMD. In this approach, known as Coulomb explosion imaging,^40,41^ molecules are ideally ionized instantaneously to a high charge state (i.e., to more than one charge per atom) so that only the Coulomb forces govern the resulting explosion and molecular binding forces can be neglected. In principle, a Coulomb-dominated scenario of this kind allows the transformation of the measured final-state momenta after the explosion back into the position space.^42^ In most cases, however, such a direct inversion is not feasible, and one instead resorts to analyzing the results in momentum space.^43,44^

If the trimer undergoes an idealized Coulomb explosion, its ions should be emitted along the three arrows labeled *p⃗*_Ne_ and *p⃗*_Kr_, i.e., at the relative emission angles α and β, as depicted in the bottom left panel of Figure 4. Assuming point charges with atomic masses fixed at the trimer ground-state equilibrium positions, classical propagation using Newton’s equations of motion yields an angle of 113° between the momentum of the neon ion and that of either krypton ion. A further observable from which geometrical properties can be obtained is the kinetic energy of the ions after the Coulomb explosion. Given the repulsive Coulomb potential, which scales as 1/*R*, the internuclear distance *R* between two singly charged atoms can be approximated (at large distances) as *R* = 1/KER (in atomic units), i.e., small internuclear distances yield a high kinetic energy release and large distances a small KER. For our current scenario of three ionic fragments, we therefore examine the relative kinetic energy between the neon and each of the krypton ions after the Coulomb explosion given as *E*_rel,Ne–Kr_ = *p⃗*_rel,Ne–Kr_^2^/(2μ), with μ being the reduced mass of neon and krypton. Consequently, if the trimer fragments from its mean ground-state geometry, we expect a relative breakup energy of *E*_rel,Ne–Kr_ = 7.16 eV. Along the same line, our idealized Coulomb explosion of the mean ground-state geometry of the trimer results in a relative emission angle β between the two krypton ions of 133° and a relative breakup energy *E*_rel,Kr–Kr_ = 4.09 eV.

**Figure 4:**
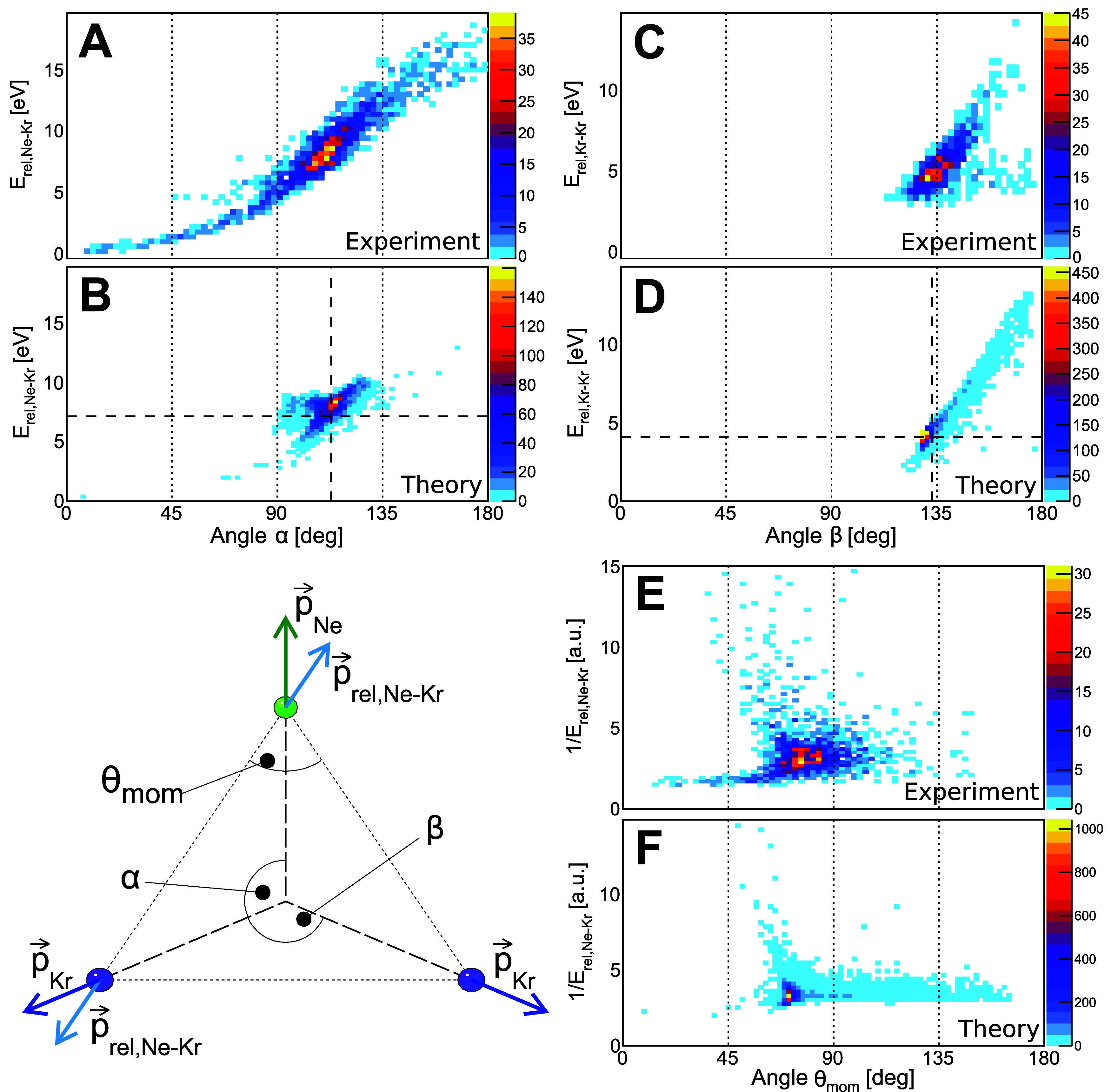
(A–D) Coincidence maps of the measured relative kinetic energy of the neon and either krypton ion and the relative emission angles α and β. (A, C) Experimental results, (B, D) corresponding plots as obtained from our simulations. The crosshairs in panels (B, D) indicate the values expected from an instantaneous Coulomb explosion of the mean ground-state geometry of the trimer. (E, F) Measured and modeled coincidence maps showing 1/*E*_rel,Ne–Kr_ as a function of the momentum-space angle θ_mom_. The color scale shows the number of measured counts.

Figure 4A,C show our corresponding experimental results, while Figure 4B,D depict the outcome of a simulated Coulomb explosion of the theoretical trimer structures after incorporating the ETMD(3) process that includes nuclear dynamics prior to decay. As before, we assume an instantaneous Coulomb explosion, treating ions as point charges with correct masses, no molecular binding, and zero initial momentum of the ions. Although being rather crude, this approximation is still expected to yield meaningful results given the large internuclear distances and the lack of chemical bond in the van der Waals cluster. The experimental data are restricted to cases where the ^1^D state was populated, i.e., the data were gated on feature 2 in Figure 3. Note that the histograms were filled twice per ETMD event in order to incorporate each of the two krypton atoms of the trimer separately. The overall agreement is good, with the experimentally observed distributions being generally less confined than those from our simulation. There are several possible sources for this, such as the experiment’s finite momentum resolution or residual background remaining even after the above-described gating. Most likely, this discrepancy originates from our simplified point-like Coulomb explosion simulation. More confined momentum-space features have been reported in earlier work on Coulomb explosion imaging;^45^ in particular, neglecting the atomic momenta of the initial state prior to the explosion is a known source of such deviations. A further contributing factor could be limitations of the computed potential energy surface, as obtaining fully dimensional, high-accuracy surfaces for such weakly bound systems remains extremely demanding. Nevertheless, the main trends are robust, and the measurements provide a stringent and valuable benchmark for theoretical approaches. Nonetheless, in both experiment and theory, the distribution of α exhibits a main feature close to the expected value of α = 113°. The same holds for the distributions of the angle β, where the main features can be found close to β = 133°. The overall broad angular emission distributions can be interpreted as initial evidence of complex, roaming-like nuclear dynamics occurring prior to ETMD. We refer to roaming-like motion as sampling shallow regions of the potential energy surface where one Ne–Kr distance is short and the other is long, resulting—as we show below—in alternating quasi-linear and triangular geometries. The measured ion kinetic energies are consistent with the expected values. The dashed crosshairs in panels (B) and (D) depict the expected values of α, *E*_rel,Ne–Kr_, β, and *E*_rel,Kr–Kr_ for the mean geometry of the ground state of the trimer.

Finally, in order to further relate our momentum-space observations to the position-space results presented below, we analyze the momentum-space angle θ_mom_ (see the sketch in Figure 4), which corresponds to the real-space angle θ (see the sketch in Figure 1), and its dependence on 1/*E*_rel,Ne–Kr_. The latter corresponds (as stated above) to the internuclear distance *R*_Ne–Kr_ between the neon and either krypton atom at the instant of ETMD(3). Angle θ_mom_ is defined as the angle between the two relative momenta of the neon atom with respect to each of the two krypton atoms. Our measured and modeled coincidence maps can be found in Figure 4E,F. The agreement between experiment and theory is good, and we indeed observe a distribution with a shape similar to that of the position-space results shown in Figure 5A. In the following section, we will present further insight into the time dependence of the roaming-like nuclear dynamics of the ionized trimer prior to ETMD(3).

**Figure 5:**
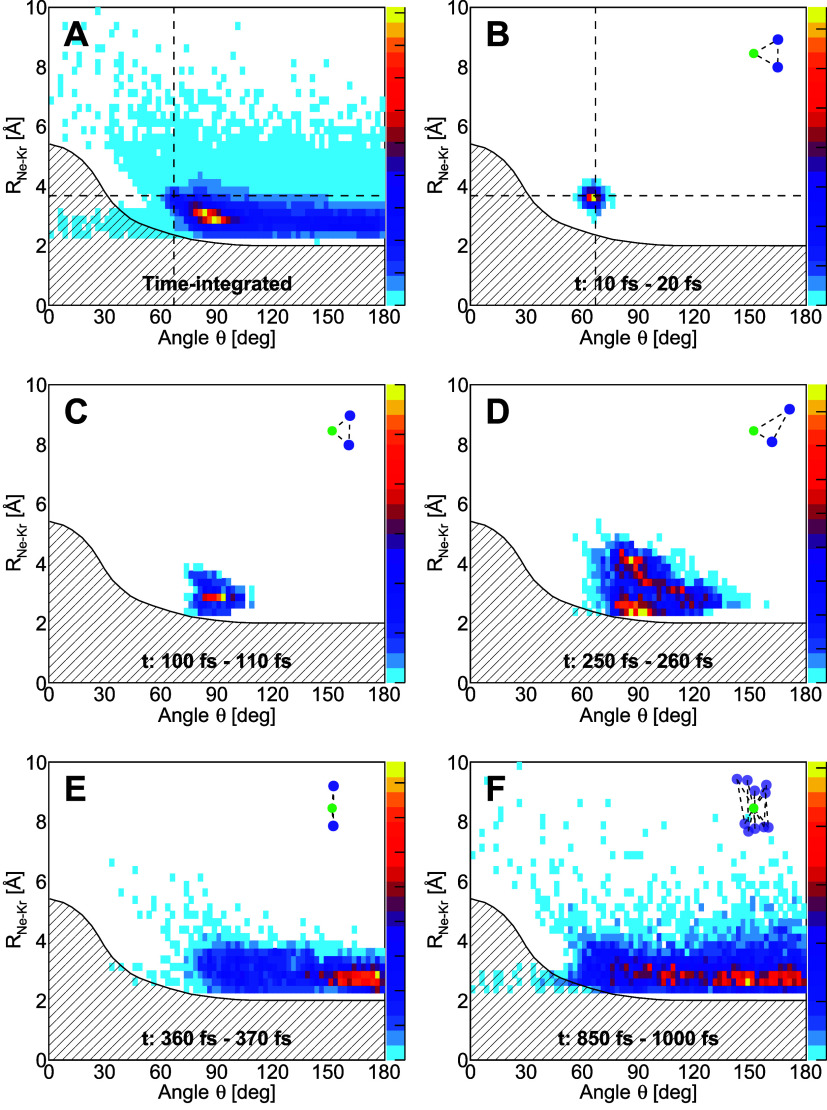
(A) Time-integrated map. (B–F) Temporal progression of the dependence of the internuclear distance *R*_Ne–Kr_ versus the opening angle θ. The panels show the geometry of the decaying trimers at certain times (indicated in each panel) after the decaying state was populated. The small insets in panels (B–F) show typical trimer geometries for the given decay time. The crosshairs in panels (A, B) indicate the mean geometry of the ground state of the NeKr_2_ trimer. The hatched area marks the region, in which a decay is energetically not possible (assuming that one of the two internuclear distances *R*_Ne–Kr_ = 3.68 Å, see text). The intensity shown as a heat map is given in arbitrary units, with intensity normalized to unity in each panel. The color scale is linear. The overall time-integrated data set shown in panel (A) consists of 70,502 trimers decaying via ETMD(3).

## III Real-Space Results and Discussion

In order to retrieve a clearer picture of the nuclear dynamics and the geometries at the instant of ETMD, we examine the results from our modeling of the ETMD(3) process in real space and real time. Figure 5A shows the corresponding results by plotting the time-integrated dependence of the internuclear distance *R*_Ne–Kr_ on the opening angle θ at the neon atom. The hatched area corresponds to the region where the dicationic initial-state potential energy surface lies energetically above the triply charged final-state potential energy surface, assuming a fixed internuclear distance of *R* = 3.68 Å between the neon and one of the two krypton atoms. In fact, we observe a contribution at values close to the mean ground-state geometry of the trimer at [θ = 67°, *R*_Ne–Kr_ = 3.68 Å], as indicated by the crosshair. However, the main contribution does not correspond to the ground-state configuration, but involves slightly shorter *R*_Ne–Kr_ values and larger opening angles θ, around 90°. Overall, the distribution covers a wide range of opening angles θ and internuclear distances up to 10 Å. Some intensity extends into the hatched region. This contribution is possible in cases where the distance between the neon atom and each of the two krypton atoms deviates from 3.68 Å.

In order to gather further information on the origin of the distribution of real-space geometries of the decaying trimer, we finally inspect the temporal evolution of the dicationic state by examining our simulation data for distinct decay times. Figure 5B–F show the corresponding results in the same representation as that of Figure 5A for different times after excitation of the dicationic state. At the shortest times (Figure 5B), only geometries close to the ground-state configuration (as indicated again by the crosshair) contribute. As time evolves, initially the angle θ increases and the internuclear distance *R*_Ne–Kr_ decreases slightly [panel (C)]. After ∼250 fs, two features emerge in Figure 5D. These belong to cases where one of the krypton atoms is substantially closer to the neon than the other. This configuration corresponds well to the intuitive picture of the ETMD process as sketched in Figure 1D: In ETMD one of the krypton atoms donates an electron to the neon ion. For this electron transfer to be efficient, the two atoms need to be sufficiently close to each other. The energy that is freed by the electron exchange is then transferred to the other krypton atom. Because this energy transfer does not rely on orbital overlap, it can still occur efficiently at much larger distances, thus favoring the observed configuration of one close and one distant krypton atom. Figure 5E (corresponding to decay times of approximately 360 fs) is strongly dominated by almost linear trimer geometries. There, the neon atom moved between the two krypton atoms, showing traces of the aforementioned pendular motion. Finally, at the longest decay times, the trimer has contracted relative to its initial ground-state configuration, and these late-time decay events span a wide range of geometries, from triangular to linear, as depicted by Figure 5F. At these longest times, ETMD(3) events associated with the largest internuclear distances also occur.

## IV Conclusions

We have performed a detailed investigation of ETMD(3) after initial neon K-shell ionization of a NeKr_2_ trimer. Our experimental results confirm the time-integrated outcome of our theoretical modeling, indicating complex nuclear dynamics occurring prior to the decay, with most trimers decaying in a molecular geometry that differs from the ground state one. Our calculations provide access to the temporal evolution of these molecular dynamics. Thus, our combined study enables us to follow the intricate dynamics of the process in real space and time; we demonstrate that a complex, roaming-like motion of the atoms of the loosely bound trimer is triggered in the excited state prior to ETMD. Our results suggest a pendular motion of the neon atom moving between the two krypton atoms, as well as configurations in which an entity consisting of the neon atom and one of the krypton atoms is formed, which is orbited by the second krypton atom. This second configuration yields ETMD at the largest internuclear distances. This is possible because one of the two krypton atoms is closer to the neon atom, allowing the efficient donation of an electron. The energy transfer that occurs as a result of the donation takes place over much larger distances. Altogether, our results showcase the detailed interplay between electronic and nuclear degrees of freedom in weakly bound systems and highlight the crucial role of time-dependent nuclear geometries in modeling nonlocal decay mechanisms. More broadly, this work demonstrates how nonlocal electronic decay processes like ETMD(3) can be employed to image the intriguing molecular dynamics of excited, loosely bound matter. Our detailed study of a prototype system serves as a benchmark for probing radiation damage pathways in complex environments such as biological or condensed-phase systems. These benchmarks enable hierarchical strategies, in which fragment-level ETMD widths are embedded within QM/MM (quantum-mechanics/molecular-mechanics) frameworks, while long-range ETMD(3) is described using the asymptotic 1/*R* ^6^ expression employed here.

## V Experimental Section

We investigated the decay dynamics described above by combining the data from two experimental campaigns. Measurements were performed at the soft X-ray beamlines U49–2_PGM-1 ^46^ and P04 ^47^ at the synchrotron-radiation facilities BESSY II (Berlin, Germany) and PETRA III (Hamburg, Germany) during single-bunch or few-bunch operation, respectively, using a cold target recoil ion momentum spectroscopy (COLTRIMS) reaction microscope.^31–33^ In brief, the synchrotron beam was crossed at right angles with a supersonic gas jet consisting of a mixture of Ne and Kr gas, generating a well-defined interaction volume defining the interaction volume for initial photoionization. The photon energy was set to 880 eV, i.e., approximately 10 eV above the neon K-shell ionization threshold.^35^ Initial photoionization triggered in most cases a local Auger-Meitner decay, emitting a second electron (of high kinetic energy). After ETMD(3), a third, low-energy electron was emitted and the trimer fragmented into three singly charged ions. Static electric and magnetic fields were used to guide charged particles toward two large-area multiple-hit-capable microchannel plate detectors with an active area of 80 mm in diameter. Both detectors were equipped with a hexagonal delay-line anode (RoentDek HEX90)^48^ for position readout. By measuring each particle’s time-of-flight and impact position on the detector, the particle’s trajectory inside the spectrometer volume was reconstructed in an offline analysis, yielding the final-state momentum vectors. From the vector momenta, we obtained angular emission directions and derived quantities such as the particle’s kinetic energy. As we measured all particles in coincidence, we also retrieved quantities such as relative emission angles between particles and sum kinetic energies from our measured data set. For the measurements at BESSY II, the following spectrometer settings were used: The electron arm consisted of a 70 mm long acceleration region and a 140 mm long field-free drift region, and the ion arm consisted of a 50.3 mm long acceleration region. We used a homogeneous electric field of 17.1 V/cm and a homogeneous magnetic field of 5.3 G. The measurements at PETRA III were performed using an electron arm consisting of a 170 mm long acceleration region and an ion arm consisting of a 120 mm long acceleration region. The strength of the electric acceleration field was 30.8 V/cm and the superimposed homogeneous magnetic field had a strength of 6.8 G. With these settings, we were able to detect all ionic fragments (Ne^+^, Kr^+^, and Kr^+^) with full 4π solid-angle coverage in the laboratory frame and electrons up to a kinetic energy of approximately 20 eV. In most cases, the high-energy Auger electron was not detected as a result of its very small solid-angle coverage.

The NeKr_2_ trimers were produced by expanding a mixture of 97.5% Ne and 2.5% Kr through a cooled nozzle of 60 μm diameter at a temperature of 140 K and a driving pressure of 4 bar. This resulted in a small fraction of NeKr_2_ trimers in the gas jet (which could nevertheless be examined via the coincidence measurement approach). The trimers in the target gas jet are in the vibrational ground state. To avoid partial clogging of the cooled nozzle due to impurities in the gas line and therefore different parameters for trimer formation, we used an active carbon filter in the gas line, which we placed in dry ice (solid form of carbon dioxide, temperature of −78.5 °C at 1 bar) to freeze out impurities like water. The supersonic gas jet passed two skimmers (0.3 mm diameter each) and was crossed with the linearly (BESSY II) or circularly (PETRA III) polarized photon beam inside our spectrometer as described above.

Valid events of ETMD(3) are identified in the experiment by performing several checks and restrictions on the measured data set. First, only photoionization events that resulted in the detection of three ions (i.e., Ne^+^, Kr^+^, and Kr^+^) and up to two electrons are considered. In order to distinguish real events from background or false coincidences, the sum momentum of the three measured ions is computed. The sum momentum corresponds to the velocity of the center of mass of the trimer in the laboratory frame and the recoil of the emitted electrons and the absorbed photon. The former contribution is removed in our data analysis by inspecting the data in the center-of-mass frame (i.e., by subtracting a fixed momentum corresponding to the jet velocity in our experiment). The latter two contributions are dominated by the recoil of the emitted Auger electron of |*p⃗*_Auger_| ∼ 7 a.u. Accordingly, filtering the measured data for events with a sum momentum |*p⃗*_sum_| < 15 a.u. leads to a strong suppression of background and false coincidences. As indicated in the main text, the momentum-space results presented in Figure 4 are further filtered by gating on the sum of the KER and the electron kinetic energy. Figure 6 shows the corresponding gate, which filters out those events where an ETMD(3) electron was detected after initial population of the Ne^2+^(^1^D) state after Ne K-shell photoionization and local Auger-Meitner decay. In addition to the gate shown there, the data is furthermore restricted to KER > 10.5 eV.

**Figure 6:**
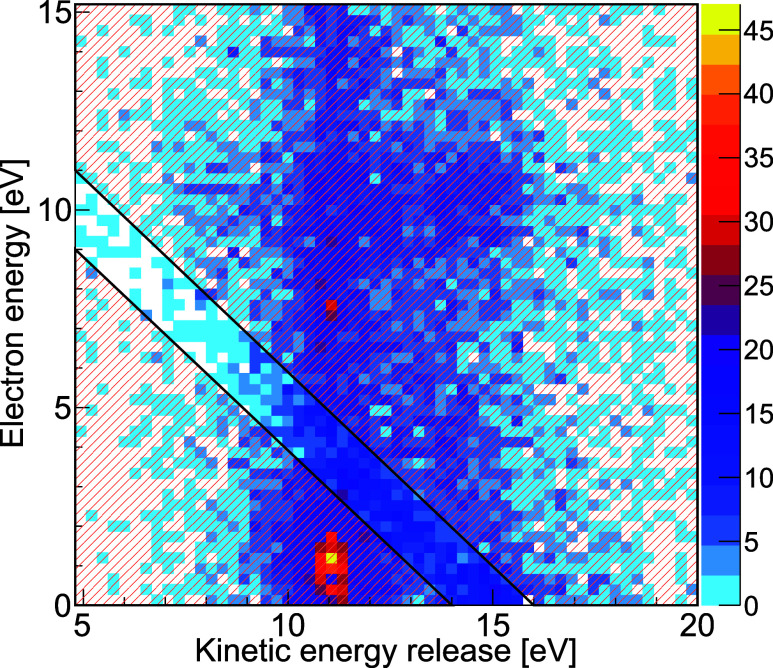
Ion momentum-space results shown in Figure 4 are restricted to cases where the sum of the kinetic energy release and the electron energy is within the range indicated in this figure. An additional gate (not shown) restricts the data set further to events where KER > 10.5 eV. See text for details.

### V.I Momentum-Space Observables

The relative kinetic energy shown in Figure 4A–D is obtained by computing the relative momentum of ion pairs within the trimer. We obtain the relative momentum between the neon and either of the krypton ions from the laboratory-frame momenta *p⃗*_Ne_ and *p⃗*_Kr_ as

$${\mathrm{p⃗}}_{rel,\mathrm{Ne}\text{−}Kr}=\frac{{\mathrm{p⃗}}_{Ne}-{\mathrm{p⃗}}_{Kr}}{2}$$

This momentum vector points (as depicted in the sketch in Figure 4) to a good approximation along the bond between the Ne and Kr atoms. The fraction of the overall breakup energy along this direction is expressed as the relative kinetic energy as

$$E_{\mathrm{rel},\mathrm{Ne}\text{−}\mathrm{Kr}}=\frac{{\mathrm{p⃗}}_{\mathrm{rel},\mathrm{Ne}\text{−}\mathrm{Kr}}^{2}}{2\mu }$$

where μ is the reduced mass of the two atoms. The corresponding relative momentum and energy between the two krypton ions Kr_A_ and Kr_B_ is given as

$${\mathrm{p⃗}}_{\mathrm{rel},\mathrm{Kr}\text{−}\mathrm{Kr}}=\frac{{\mathrm{p⃗}}_{Kr_{A}}-{\mathrm{p⃗}}_{Kr_{B}}}{2}$$

and

$$E_{\mathrm{rel},\mathrm{Kr}\text{−}\mathrm{Kr}}=\frac{{\mathrm{p⃗}}_{\mathrm{rel},\mathrm{Kr}\text{−}\mathrm{Kr}}^{2}}{2\mu }$$

## VI Theory

To describe the ETMD(3) process in NeKr_2_, we first computed the fully dimensional nuclear dynamics on the dicationic potential energy surface (PES) of the dominantly populated Ne^2+^(2p^–2 1^D) state of the NeKr_2_ cluster. This dicationic PES was calculated *ab initio* by adding the double ionization potential to the ground-state NeKr_2_ PES. The latter was obtained using the CCSD­(T) method implemented in the GAMESS US (v. 2019 R2) quantum chemistry package.^49^ For both Kr and Ne atoms, aug-cc-pVQZ correlation-consistent basis sets^50,51^ located on the corresponding atoms were used. The double ionization potential was calculated *ab initio* using the algebraic diagrammatic construction scheme for the two-particle propagator [ADC(2)].^52,53^ CCSD­(T) provides accurate ground-state energetics, while ADC(2) yields reliable double ionization potentials at moderate computational cost and has been extensively validated for noble-gas clusters. The equilibrium geometry of the decaying Ne^2+^(2p^–2 1^D) state possesses *D*_∞*h*_ symmetry with an interatomic Ne–Kr distance of about 2.7 Å. The energies of the final Ne^+^(2p^–1^)·[Kr^+^(4p^–1^)]_2_ states are represented by a single PES, and the spin–orbit splitting of the cation Kr^+^(4p^–1 2^P) was not taken into account; instead, the weighted average *I*_Kr_ = 14.22 eV was used. The tricationic PES was calculated by adding to the ground-state potential energy of NeKr_2_ the triple ionization potential, which is dominated by long-range Coulomb interactions and was approximated analytically as *I*_Ne_ + 2*I*_Kr_ + 1/*R*_1_ + 1/*R*_2_ + 1/*R*_3_, where *R*_1_ and *R*_2_ are the interatomic distances of Ne–Kr and *R*_3_ is the distance of Kr–Kr. Here, *I*_X_ denotes the ionization potential of the atom X (Ne or Kr).

Rotational motion was neglected, and the trimer was treated in its rotational ground state. Classical nuclear dynamics in the valence coordinates (*R*_1_, *R*_2_, θ) were propagated on the Ne^2+^(2p^–2 1^D, *b*_1_) dicationic PES. Five thousand trajectories were sampled from the neutral NeKr_2_ vibrational ground-state wave function; positions were drawn from this wave function, and conjugate momenta were assigned as independent zero-mean Gaussians with variances σ_*p*_*i*__^2^ = ℏ^2^/(4σ_*q*_*i*__^2^) (Wigner-like). Trajectories were integrated using a fourth-order Runge–Kutta scheme up to 3 ps. The ETMD(3) rate, Γ_ETMD(3)_(*R*_1_, *R*_2_, θ), was evaluated approximately every 7 fs along each trajectory and is defined below.

We use ETMD(3) rates Γ_ETMD(3)_ calculated using the *ab initio* Fano-ADC-Stieltjes method^29,54^ for short-range (2–4 Å) symmetric configurations for the state of *b*_1_ symmetry, where both orbitals with holes lie in the plane of the trimer. For longer distances or asymmetric configurations, we employ the asymptotic formula^55^

$$\Gamma_{\mathrm{ETMD}\left(3\right)}\left(R_{1},R_{2},\theta \right)=\frac{3c^{4}}{4\pi }\left(\frac{\Gamma_{\mathrm{RCT}}\left(R_{1},\theta \right)\sigma_{\mathrm{Kr}}}{\omega_{\mathrm{vp}}^{4}R_{2}^{6}}+\frac{\Gamma_{\mathrm{RCT}}\left(R_{2},\theta \right)\sigma_{\mathrm{Kr}}}{\omega_{\mathrm{vp}}^{4}R_{1}^{6}}\right)$$

where σ_Kr_ is the photoionization cross section of Kr at the virtual photon energy ω_vp_, and Γ_RCT_(*R*, θ) is the radiative charge transfer (RCT) rate for a pair of Ne–Kr separated by *R* with the angle of Kr–Ne–Kr of θ. The asymptotic formula corresponds to the interpretation of ETMD(3) as a radiative charge transfer between the initial Ne dication and one of the Kr neighbors where the *virtual* photon transfers energy from Ne to the other Kr neighbor and ionizes it. In a standard RCT, Ne^2+^ Kr → Ne^+^ Kr^+^ + ℏω, a charge is transferred to the neighbor and a photon of energy ℏω is emitted. Rather than emitting a real photon, ETMD(3) involves the emission of a virtual photon that ionizes another neighbor. This energy-transfer step has a long-range asymptotic behavior proportional to 1/*R* ^6^, see also ref 56. The RCT rate is known to behave as Γ_RCT_(*R*) ≈ e^–κ*R*^,^39^ recalling the behavior of charge transfer in ETMD.^3^ In a truly asymptotic expression, i.e., where *R*_1_ and *R*_2_ are large, the RCT rate should only depend on the respective interatomic distance *R*. However, since the distances used here are not sufficiently large, we have also taken into account the θ dependence.

Within the semiclassical approximation, the matrix element Γ_ETMD(3)_ is replaced by Γ(R(t)) , where R(t) is a classical trajectory. At each time step Δ*t*, the probability that a trajectory transitions to the final state is evaluated as P(t)=Γ(R(t))Δt .^57^ The full details of the present calculations can be found elsewhere.^55^

## Supplementary Material





## Notes

S0: Movie of a typical roaming trajectory in real space. Example of a roaming trajectory occurring in the excited state of the trimer prior to ETMD(3). The dark blue ball corresponds to the neon atom, the two krypton atoms are shown in light brown. The decay process itself is disabled for this trajectory in order to better visualize the motion of the atoms in the decaying state over a longer period of time (MP4)
S01: Time-resolved position-space map of ETMD(3) in NeKr_2_. Movie of the temporal evolution of the Ne−Kr internuclear distance versus the opening angle θ as shown in Figure 5 of the main manuscript. See the description of Figure 5 for details (GIF)
