# Supplementary figures and images for: Tracking the Complex Dynamics of Electron-Transfer-Mediated Decay in Real Space and Time
Source: J Am Chem Soc. 2026 Jan 22;148(4):4126–35. doi: 10.1021/jacs.5c15510 (PMC12879743; doi:10.1021/jacs.5c15510)

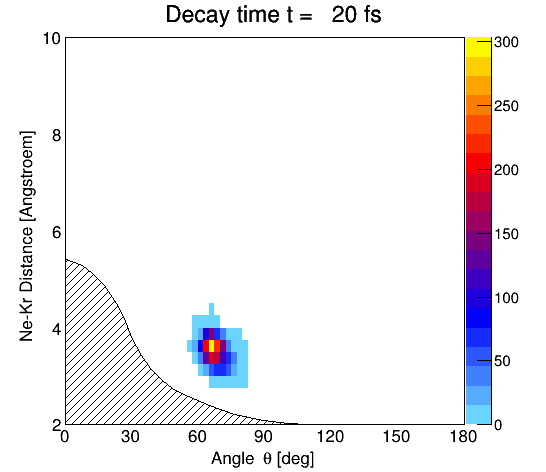

Supplement: Supplementary file 2 [file ja5c15510_si_002.gif]
